# Novel Selective and Potent EGFR Inhibitor that Overcomes T790M-Mediated Resistance in Non-Small Cell Lung Cancer

**DOI:** 10.3390/molecules21111462

**Published:** 2016-11-02

**Authors:** Yanxia Li, Zhendong Song, Yue Jin, Zeyao Tang, Jian Kang, Xiaodong Ma

**Affiliations:** 1 Institute of Respiratory Diseases, The First Affiliated Hospital of China Medical University, Shenyang 110001, Liaoning Province, China; liyanxia001@163.com; 2 Department of Respiratory Medicine, The First Affiliated Hospital of Dalian Medical University, Dalian 116011, Liaoning Province, China; 3 College of Pharmacy, Dalian Medical University, Dalian 116044, Liaoning Province, China; Songzhen@163.com (Z.S.); rutin@sina.com (Y.J.); tangzeyao@aliyun.com (Z.T.)

**Keywords:** NSCLC, EGFR T790M, resistance, pyrimidine, inhibitor

## Abstract

Treating patients suffering from EGFR mutant non-small cell lung cancer (NSCLC) with first-generation EGFR tyrosine kinase inhibitors (EGFR TKI) provides excellent response rates. However, approximately 60% of all patients ultimately develop drug resistance due to a second T790M EGFR TKI mutation. In this study, we report the novel molecule *N*-(3-((5-chloro-2-(4-((1-morpholino)methyl)phenylamino)-4-pyrimidinyl)amino)phenyl)acrylamide (DY3002) to preferentially inhibit the EGFR T790M mutant (EGFR^T790M^) (IC_50_ = 0.71 nM) over wild-type EGFR (IC_50_ = 448.7 nM) in kinase assays. Compared to rociletinib (SI = 21.4) and osimertinib (SI = 40.9), it significantly increased selectivity (SI = 632.0) against EGFR^T790M^ over wild-type EGFR. Furthermore, in cell-based tests, DY3002, with an IC_50_ value of 0.037 μM, exhibited enhanced inhibitory potency against H1975 cells. Moreover, AO/EB and DAPI staining assays as well as flow cytometer analyses indicated that DY3002 possesses superior biological properties compared to alternatives. In addition, a rat oral glucose tolerance test revealed that treatment with high drug doses (50 mg/kg) of DY3002 did not result in hyperglycemia, suggesting a reduction of side effects in NSCLC patients will be achievable relative to established EGFR inhibitors. In summary, our findings indicate DY3002 as a promising preclinical candidate for effective treatment of patients with EGFR^T790M^-mutated NSCLC.

## 1. Introduction

**Figure 1:**
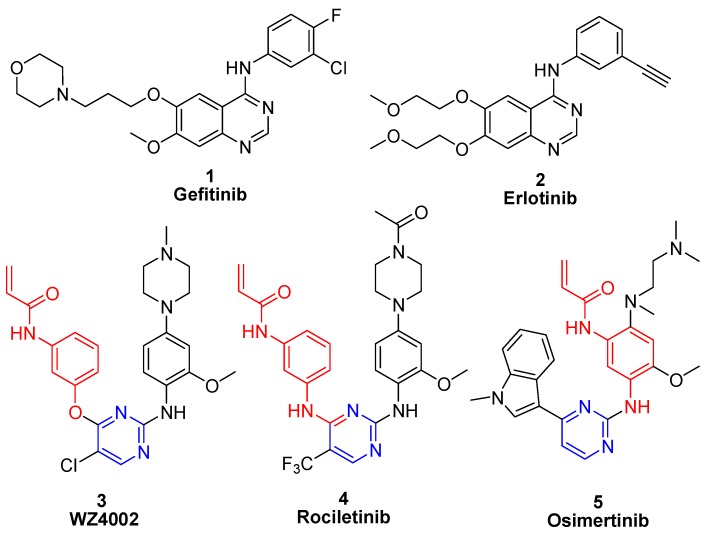
Structures of novel EGFR inhibitors.

Non-small cell lung cancer (NSCLC) accounts for approximately 85% of all lung cancers [1]. Genetic aberrations within the tyrosine kinase domain of the epidermal growth factor receptor (EGFR) have been identified as key drivers of NSCLC progression [2]. Inhibition of the EGFR kinase domain and resulting oncogenic cell signaling disruption via small molecule inhibitors, such as gefitinib (**1**) and erlotinib (**2**) (Figure 1) has been shown to be particularly beneficial for patients carrying the so-called “sensitizing mutations”, such as L858R or the exon-19 deletion [3,4,5]. Although such EGFR inhibitor drugs exhibit high response rates, clinical experiments demonstrated that approximately 60% of patients would eventually suffer from drug resistance after a treatment period, due to a conversion of the “gatekeeper” threonine 790 to methionine (T790M) [6,7,8].

**Figure 2:**
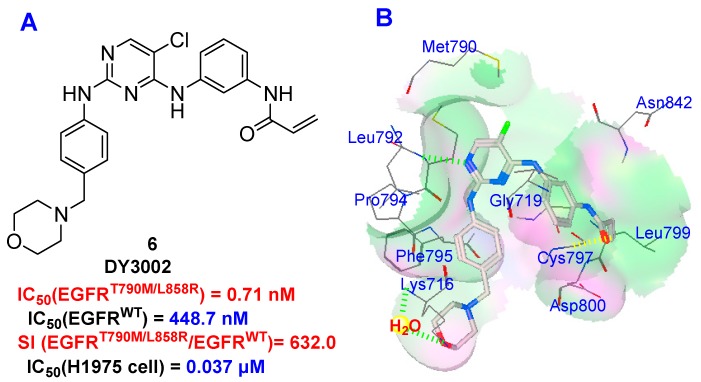
Structure of DY3002 (**A**) and its putative binding mode with EGFR^T790M^ enzyme (**B**).

Recently, irreversible EGFR inhibitors have been developed that contain a Michael acceptor functional group and that circumvent the T790M mutation-related resistance. These inhibitors form a covalent bond with Cys797 within the EGFR active site and have revealed preclinical activity against T790M-containing mutants of EGFR [9]. However, wild-type EGFR and EGFR T790M mutant share highly similar three-dimensional structures and virtually identical binding affinities with ATP. Consequently, almost all of the reported, irreversible EGFR inhibitors displayed equal potencies against T790M mutant and wild-type enzyme, highlighting the current challenge in the search for EGFR^T790M^ mutant-selective inhibitors [10,11]. WZ4002 (**3**) [12], rociletinib (**4**) [13], and osimertinib (**5**) [14,15] are typical EGFR T790M inhibitors and a phase trial has revealed capability in gefitinib-resistant NSCLC patients who suffer from EGFR mutations (Figure 1). Unfortunately, only osimertinib possesses exceptional biological properties, and has thus been approved in 2015 for the treatment of patients with metastatic EGFR T790M mutation-positive NSCLC [16]. On the basis of considerable efforts to identify more effective and safe agents to overcome gefitinib-produced resistances [17,18,19], we found the novel diphenylpyrimidine derivative *N*-(3-((5-chloro-2-(4-((1-morpholino)methyl)phenylamino)-4-pyrimidinyl)amino)phenyl)acrylamide (**6**, DY3002, Figure 2) [20]. The molecule displayed enhanced activity and increased selectivity against EGFR^T790M^ and was thus reported as a promising NSCLC clinical candidate.

## 2. Results and Discussion

### 2.1. Inhibition of Kinase and Cancer Cell Viability

**Figure 3:**
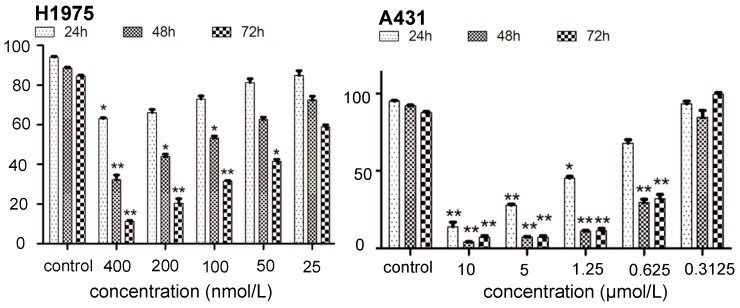
The effects of treating time and concentrations of DY3002 on cell viability. * *p* < 0.05; ** *p* < 0.01.

**Figure 4:**
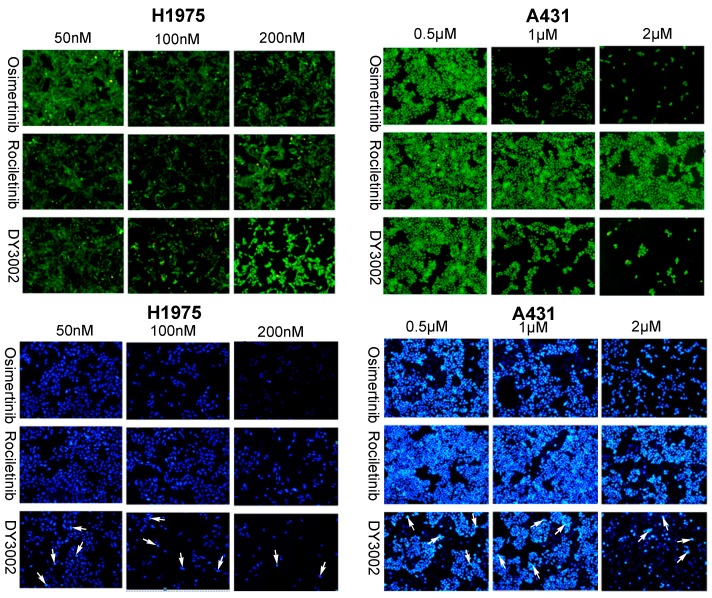
Morphological changes of H1975 and A431 cells (100×, final magnification, green images: AO/EB double fluorescent staining of H1975 and A431 cells treated with different concentrations of osimertinib, rociletinib, and DY3002 for 48 h, blue images: DAPI staining of H1975 and A431 cells treated with different concentrations of osimertinib, rociletinib, and DY3002 for 48 h). White arrows represented typical apoptotic cancer cells.

As shown in Table 1, DY3002 strongly inhibited EGFR^T790M/L858R^ enzyme activity at a surprisingly low concentration of 0.71 nM. In contrast, high concentrations (460.2 nM) are necessary to interfere with wild-type EGFR. Thus, DY3002 possesses a 632.0-fold higher selectivity against EGFR^T790M/L858R^ mutants that against wild-type EGFR, suggesting that it will reduce side effects, such as the hyperglycemia problem. Compared to rociletinib (SI = 21.4) and osimertinib (SI = 40.9), DY3002 features approximately 15- and 30-fold higher selectivity, respectively. Motivated by this excellent activity, DY3002 was also evaluated in vitro for activity against a series of lung cancer cell lines, including H1975^T790M^, HCC827^del E746_A750^, A431^wild-type^, H1299^wild-type^, and A549^wild-type and k-ras^. Additionally, normal human bronchial cell line (HBE) and normal liver cell line (LO-2) were also tested to explore cytotoxicity on cellular level using CCK-8 assay method. The results are presented in Table 2 and indicate that DY3002 features strong activity (IC_50_ = 0.037 μM) against H1975 cells with EGFR^T790M^ compared to reference compounds. Yet, it only moderately inhibited proliferations of the wild type cell lines A431, H1299, LoVo, and A549 with IC_50_ values of 0.382 μM, 4.12 μM, 2.46 μM, and 4.45 μM, respectively. For activity against HCC827 cells with EGFR del E746_A750, all these inhibitors are potent, with IC_50_ values of approximately 0.010 μM. Fortunately, DY3002 is not sensitive to normal cell lines LO-2 (IC_50_ = 4.24 μM) and HBE (IC_50_ > 40.0 μM), suggesting low cell cytotoxicity. Figure 3, reveals remarkable inhibition of A431 cells and H1975 cells proliferations increasing DY3002 concentrations with a range from 25 to 400 nmol/L, and 0.3125 μM to 10 μM, respectively. AO/EB and DAPI staining assays were carried out to investigate the apoptotic morphology of cancer cells. As shown in Figure 4, subsequent to AO/EB or DAPI staining, both EGFR^T790M^-mutated H1975 cells and wild-type EGFR-mutated A431 cells that were exposed to DY3002 revealed striking nuclear condensation, membrane blebbing, nuclear fragmentation, and apoptotic bodies, all of which are characteristics of apoptotic programmed cell death.

### 2.2. Wound Healing and Transwell Assays

**Figure 5:**
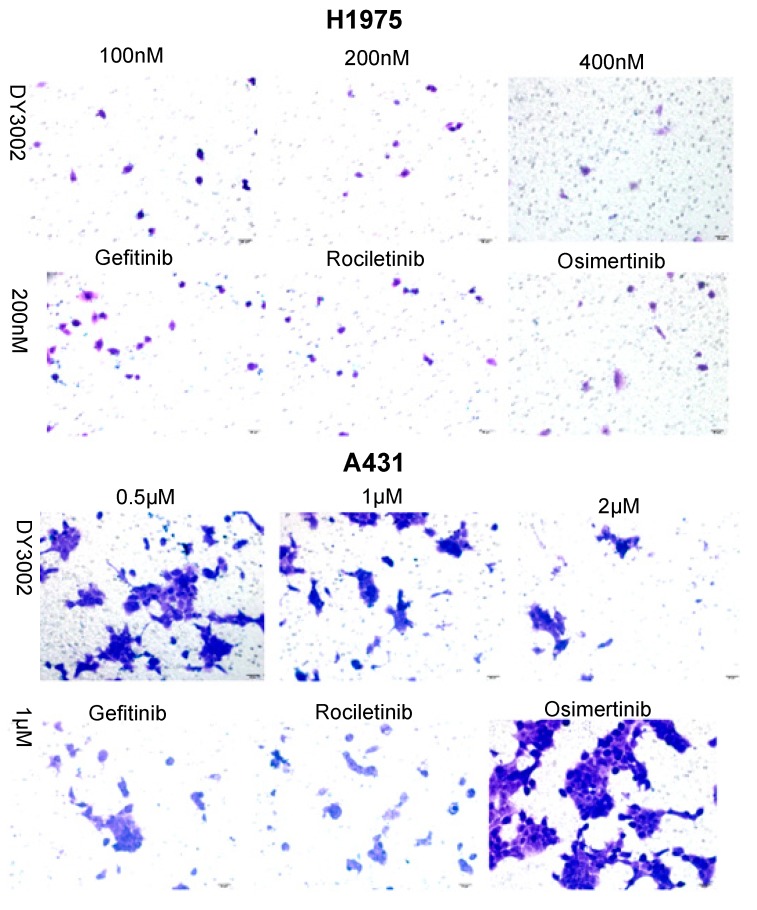
Representative images of H1975 and A431 cells treated with DY3002, rociletinib, and osimertinib by the transwell invasion assay.

**Figure 6:**
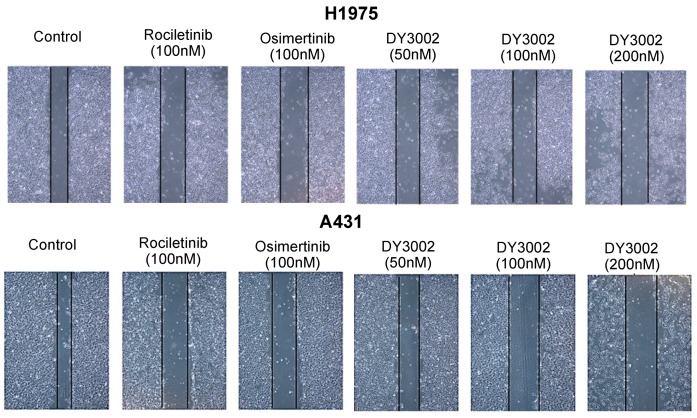
Representative images of H1975 and A431 cells treated with different concentrations of DY3002, rociletinib, and osimertinib for 48 h by the wound-healing assay.

To determine if DY3002 can prevent A431 and H1975 cell migration, wound healing and transwell assays were performed. As indicated in Figure 5 and Figure 6, DY3002 could strongly inhibit migration of H1975 cells, but only moderately interfered with A431 cells. Below concentrations of 200 nM, DY3002 is equivalent to osimertinib and rociletinib in inhibiting the migration of H1975 cells; however, interference with A431 cells is weaker compared to osimertinib. These results are in accordance with explorations based on CCK-8 assays, indicating a slightly lower selectivity of DY3002 compared to osimertinib against resistant H1975 cells over wild-type A431 cells.

### 2.3. Effects on ROS Levels

**Figure 7:**
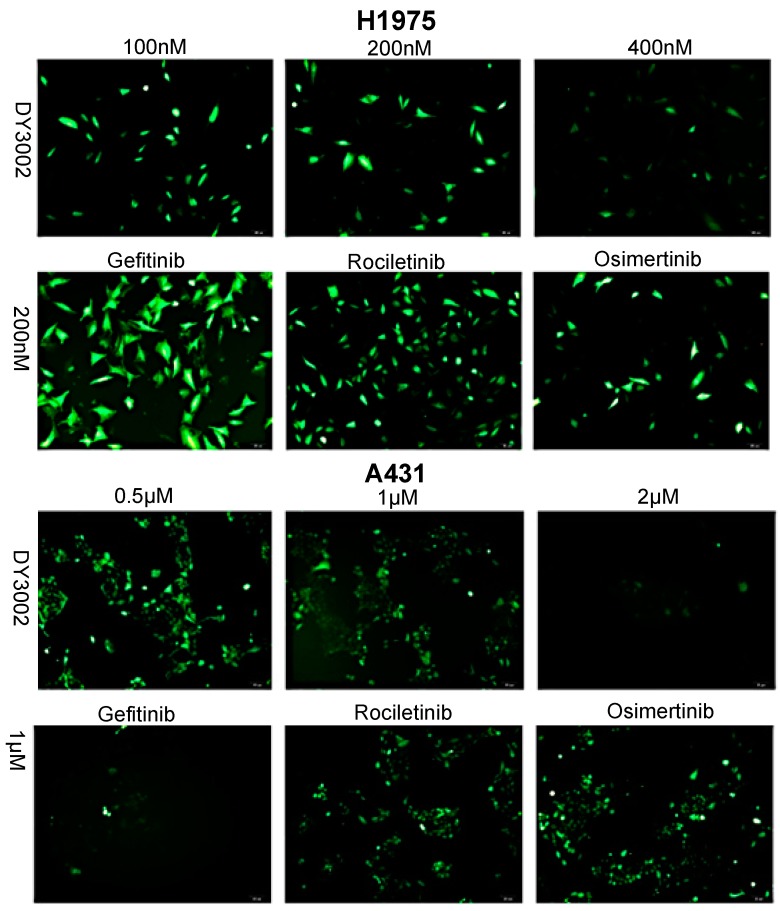
Representative images of H1975 and A431 cells treated with DY3002, rociletinib, and osimertinib by the ROS detection. Cells were treated with different concentrations of inhibitors for 24 h, then the cells were harvested, resuspended in 1 mL of DCFH-DA (10 mM), and detected the levels of ROS.

An emerging theme in signal transduction research via growth factor receptors and adhesion molecules is the role of reactive oxygen species (ROS). Hydrogen peroxide (H_2_O_2_) in particular is an essential downstream intermediate, modulating cell responses via transient and reversible oxidation of key intracellular signaling components [21]. Consequently, DY3002 had an effect on ROS generation in H1975 and A431 cells (Figure 7). In fact, DY3002, rociletinib, and osimertinib effectively inhibited ROS generation in both A431 and H1975 cells. Moreover, this assay revealed that all of the tested treatment options are more sensitive to H1975 cells with T790M mutation than to wild-type A431 cells.

### 2.4. Flow Cytometer Analysis

**Figure 8:**
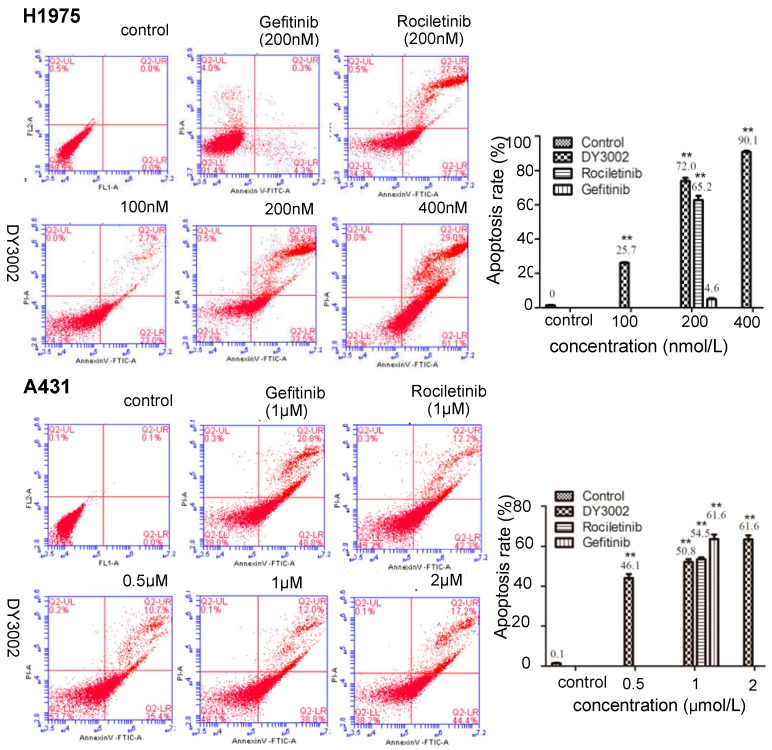
Effects of DY3002, rociletinib, and gefitinib on A431 and H1975 cells cycle arrest detected by flow cytometry assay. Cells were treated by different concentrations of inhibitors for 48 h, then were stained with Annexin V-FITC/propidium iodide and analyzed by flow cytometry assay. Results are representative of three separate experiments, dates are expressed as the mean ± standard deviation, ** *p* < 0.01.

As shown in Figure 8, DY3002 triggered apoptosis in EGFR^T790M^-mutated H1975 cells in a dose- and time-dependent manner. Apoptosis rates remarkably increased from 25.7% to 90.1% in H1975 cells treated with DY3002 (100 nM, 200 nM, and 400 nM) for 48 h. At identical concentration (200 nM), the apoptosis rate of DY3002 (72.0%) was slightly higher compared to rociletinib (65.2%), while it is much higher compared to gefitinib (4.6%). For A431 cells, the percentages of apoptotic cells clearly increased from 46.1% to 61.6% via DY3002 treatment (0.5 μM, 1 μM, and 2 μM) for 48 h. Compared to gefitinib (61.6%), both DY3002 (50.8%), and rociletinib (54.5%) possess decreased apoptosis rates.

**Figure 9:**
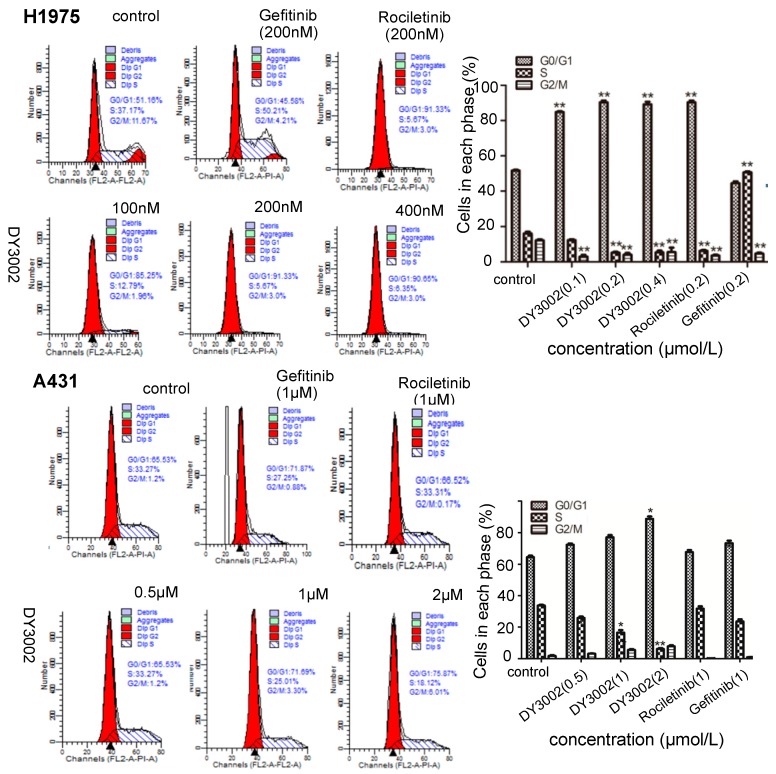
Effects of DY3002, rociletinib, and gefitinib on H1975 and A431 cells cycle arrest detected by flow cytometry assay. Cells were treated with different concentrations of inhibitors for 48 h, collected and fixed with 70% ethanol at 4 °C overnight. Then, the cells were stained by the mixture containing 5 mL propidium iodide for 10 min at 37 °C, and the cell cycle was analyzed by a flow cytometer. * *p* < 0.05; ** *p* < 0.01.

To investigate the effects of DY3002 on cell-cycle progressions in H1975 and A431 cells, we measured DNA content of cancer cells that were treated with DY3002 and reference compounds using a flow cytometer. Representative diagrams are shown in Figure 9. Evidently, DY3002 significantly locked H1975 cells at the S phase. Compared to control group, the percentages of the G0/G1 phase increased from 51.16% to 91.33%, and those of the S phase decreased from 37.17% to 5.67% via treatment with DY3002 at concentrations from 100 nM, 200 nM, and 400 nM for 48 h. However, the percentages of the G2/M phase had only minor changes. For A431 cells, the proportion of the G0/G1 phase increased from 65.53% to 75.87% subsequent to treatment of the cancer cells with DY3002 (0.5 μM, 1 μM, and 2 μM) for 48 h, revealing that DY3002 could cause a G0/G1 arrest in A431 cells.

### 2.5. Molecular Simulation

In addition, DY3002 was docked into the ATP-binding site in a model of EGFR kinase with T790M mutation (PDB code: 3IKA) to explore its putative interaction mechanism [12]. We applied AutoDock 4.2 in parallel with default parameters [22,23]. The results are shown in Figure 2B, revealing DY3002 to form several strong interactions with EGFR^T790M^, including: (1) a covalent bond between the acryl amide functionality with the amino acid Cys797; (2) a strong contact generated from the chlorine atom at the *C*-5 position of the pyrimidine core with mutant gatekeeper residue Met790; (3) strong hydrogen bonds between the *N*-1 nitrogen atom of the pyrimidine core and the amino acid Leu792, as well as the oxygen atom of the morpholine ring with Lys716 through a water molecule. Through these strong contacts, DY3002 robustly interacts with the EGFR^T790M^ enzyme, in accordance with the observed strong anti-EGFR^T790M^ activity.

### 2.6. In Vivo Blood Sugar Test

**Figure 10:**
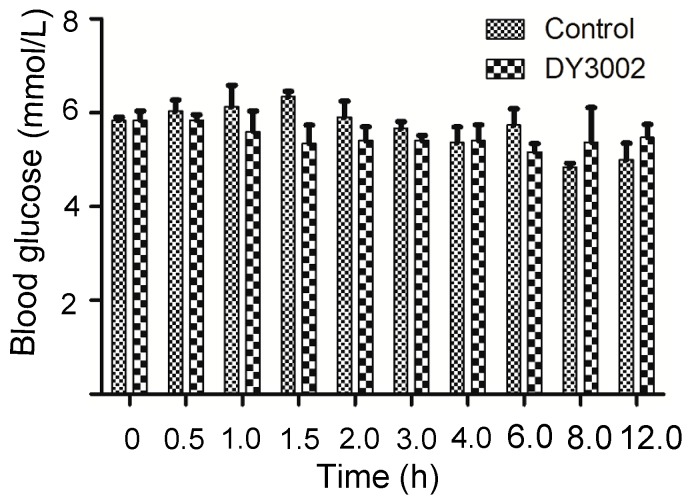
The effects of DY3002 on the blood glucose concentration in a rat model.

As reported, rociletinib potentially produces a major side effect of hyperglycemia when administered in high dose to patients during clinical trails [24,25]. To confirm whether DY3002 will produce this effect in rats as well, the experiment is simply assessing glucose tolerance in healthy rats for which no positive control was performed. Rates were orally given compound DY3002 (HBr form, 50 mg/kg) and a vehicle for comparison. Figure 10 confirms that DY3002 did not yield hyperglycemia compared to control group at high drug doses in healthy rats, indicating low toxicity and consequent suitability for further development.

## 3. Materials and Methods

### 3.1. Cell Culture and Reagents

H1975, HCC827, A549, and H1299 human NSCLC adenocarcinoma cells were obtained from the American Type Culture Collection. A431 human epidermoid carcinoma cells were kind gifts from Fuheng Biology Company (Shanghai, China). LoVo human colon carcinoma cells, HBE human bronchial epithelioid cells, LO-2 human normal hepatocyte cells were kind gifts from YuXi Biotech Company (Jiangsu Province, China). The Cell Counting Kit-8 (CCK-8) reagent was obtained from Biotool Company (Kirchberg, Switzerland). The wild-type EGFR enzyme, mutant EGFR L858R/T790M enzyme, and the ADP-Glo™ Kinase Assay system that measures ADP formed from a kinase reaction were purchased from Promega Corporation (Fitchburg, WI, USA). The Annexin V-FITC Apoptosis Detection Kit and Cell Cycle Assay were purchased from Beyotime Company (Shanghai, China). H1975, HCC827, and H1299 cells were grown in RPMI-1640 (Gibco^®^, Big Cabin, OK, USA) supplemented with 10% FBS (Gibco^®^) and 1% penicillin-streptomycin (Beyotime Company, Shanghai, China). A431, LoVo, HBE, and LO-2 cells were grown in DMEM (Gibco^®^) supplemented with 10% FBS (Gibco^®^) and 1% penicillin-streptomycin (Beyotime Company, Shanghai, China). All cells were maintained and propagated as monolayer cultures at 37 °C in humidified 5% CO_2_ incubator.

### 3.2. Statistical Analysis Data

Significant differences among groups were analyzed using the one-way ANOVA test. Duncan’s multiple range test was used to determine the statistical significance (*p* < 0.01 and *p* < 0.05) between control and DY3002-treated groups. All statistical analyses were performed with SPSS 17.0 software (SPSS Inc., Chicago, IL, USA).

### 3.3. Biological Test Method

#### 3.3.1. Kinase Enzymatic Assays

The wild-type EGFR kinase enzyme system (Catalog. V3831) and the T790M/L858R-mutated EGFR kinase enzyme (Catalog. V5324) were purchased from Promega Corporation (Fitchburg, WI, USA). Concentrations consisting of suitable levels from 0.1 to 100 nM were used for all of the tested compunds. The experiments were performed according to the instructions of the manufacturer. The more detailed and complete protocols, see the ADP-Glo™ kinase Assay Technical Manual #313, and the active kinase datasheet available at: http://www.promega.com/tbs/tm313/tm313/tm313.html and http://www.promega.com/KESProtocol (or http://www.promega.com/tbs/signaling.htm), respectively. The test was performed in a 384-well plate, and includes the major steps below: (1) perform a 5 μL kinase reaction using 1× kinase buffer (e.g., 1× reaction buffer A); (2) incubate at room temperature for 60 min; (3) add 5 μL of ADP-Glo™ Reagent to stop the kinase reaction and deplete the unconsumed ATP, leaving only ADP and a very low background of ATP; (4) incubate at room temperature for 40 min; (5) add 10 μL of Kinase Detection; (6) reagent to convert ADP to ATP and introduce luciferase and luciferin to detect ATP; (7) incubate at room temperature for 30 min; (8) plate was measured on TriStar^®^ LB942 Multimode Microplate Reader (BERTHOLD TECHNOLOGIES GmbH & Co. KG., Bad Wildbad, Germany) to detect the luminescence (Integration time 0.5–1 s). Curve fitting and data presentations were performed using GraphPad Prism version 5.0 (GraphPad Software, Inc.).

#### 3.3.2. Cell Growth Inhibitory Activity

##### CCK-8 Assay

All the cell viability assays were performed according to the CCK-8 method. The cells were seeded at a density of 5 to 8 × 10^4^ cells/mL in 96-well plates in growth medium supplemented with 10% serum at 37 °C with 5% CO_2_ for one day. After 12 h of incubation, 100 μL of medium was removed, and 100 μL of sample solution with different concentrations of inhibitor was added and then the cells were incubated for 48 or 72 h. Subsequently, 10 μL of CCK-8 reagent (Biotool Company, Kirchberg, Switzerland, 5.0 mg/mL) dissolved in phosphate-buffered saline (PBS) was added and the cells were incubated for another 4 h. The absorbance was read at 450 nm with a microplate reader (Thermo Fisher Scientific, Waltham, MA, USA). The data were calculated using GraphPad Prim version 5.0.

##### AO/EB and DAPI Staining Assay

Approximately 2 × 10^5^ cells/well of A431 and H1975 cells in 6-well plates were incubated in an incubator for 48 h, then treated with different concentrations of inhibitors for 48 h. After incubation, the cells were washed with PBS twice. Then, total of 20 μL of the solution containing the AO/EB dye mix (1.0 μg/mL of AO and 1.0 μg/mL of EB in PBS) was added to the cells. The apoptotic, necrotic, and live cells were observed and counted under the fluorescent microscope (OLYMPUS, Tokyo, Japan). DAPI staining was performed after being treated as mentioned above. The cells plated in 6-well plates were washed twice with PBS and fixed with 10% formaldehyde for 10 min, then washed with PBS three times. Cells were subsequently incubated in DAPI (1.0 μg/mL) solution at room temperature for 10 min, washed with PBS and examined under a fluorescence microscope (OLYMPUS).

#### 3.3.3. Wound-Healing Assay

The cancer cells were cultured in 6-well plates for 48 h at 37 °C. Wounds were created in the cell monolayer and washed with PBS to remove cell debris, then the cells were treated with different concentrations of inhibitor for 48 h. After that, the dead cells were washed away with PBS, and the images were taken by the fluorescence microscope (OLYMPUS).

#### 3.3.4. Transwell Invasion Assay

Cell migration assay was evaluated by using Boyden chambers containing a transwell membrane filter with an 8 μm size pore (Corning Costa Corp, Cambridge, MA, USA). Prior to the invasion assay, the filter membrane was coated with 60 μL of Matrigel (BD Biosciences, Billerica, MA, USA) at a 1:8 dilution and rehydrated by adding 0.5 M serum-free medium to the apical side of the chamber at 37 °C for 0.5 h. The cells (2 × 10^5^ cells/well for invasion assay) were seeded to the apical side of the chamber with 200 μL medium with different concentrations of inhibitors, and the basolateral side of the chamber was filled with 600 μL medium containing 10% FBS. After 24 h at 37 °C, the cells adherent to the upper surface of the filter were swept by cotton swabs, then fixed with methanol, stained with crystal violet, and the cells were counted under a microscope in five random fields, irrespective of staining intensity. The images were taken by the fluorescence microscope (OLYMPUS).

#### 3.3.5. Detection of Intracellular ROS Generation

The human H1975 and A431 cells at a density of approximately 2 × 10^5^ cells/well were plated in a 6-well plate and then treated with different concentrations of inhibitors for 24 h. Then the cells were harvested, resuspended in 1 mL of DCFH-DA (10 mM), and the levels of ROS were determined by flow cytometry (Becton-Dickinson, Franklin Lakes, NJ, USA).

#### 3.3.6. Cell Apoptosis

The H1975 and A431 cells (1 to 5 × 10^5^ cells/well) incubated in 6-well plates were treated by different concentrations of inhibitors for 48 h. Then, they were collected and fixed with 70% ethanol at 4 °C overnight. After beening fixed with 75% ethanol at 4 °C for 24 h, the cells were stained with Annexin V-FITC (5 μL)/propidium iodide (5 μL) and analyzed by flow cytometry assay (Becton-Dickinson, Franklin Lakes, NJ, USA).

#### 3.3.7. Cell Cycle Analysis

The H1975 and A431 cells at a density of approximately 2 × 10^5^ cells/well were incubated in 6-well plates, treated with different concentrations of inhibitors for 48 h, collected and fixed with 70% ethanol at 4 °C overnight. After fixation, the cells were washed with PBS and stained with propidium iodide (PI) for 10 min under subdued light. Stained cells were analyzed by flow cytometry assay (Becton-Dickinson, Franklin Lakes, NJ, USA).

#### 3.3.8. Glucose Concentration Determination

The Roche Accu-chek^®^ Go blood meter was used for the determination of glucose concentration. Before testing, standard set-up of blood meter was performed according to the instrument’s specifications. The meter displays the blood sugar level in units of mmol/L. Male SD rates (180–220 g) were fasted overnight prior to dosing the next morning. Water was allowed ad libitum throughout the study. Rats were orally given compound DY3002 (50 mg/kg) and a vehicle. For the oral study, the compounds were formulated as a solution (Solutol HS15: NS = 30:70, *v*:*v*). Sampling occurred prior to dosing and at 7–9 different time points up to 12 h. Concentration of glucose in the blood was determined by blood meter.

### 3.4. Molecular Modeling

Docking studies were carried out on AutoDock 4.2 (The Scripps Research Institute, MB-5, y, La Jolla, CA, USA). The crystal structure (PDB: 3IKA) of the kinase domain of EGFR^T790M^ bound to inhibitor **3** was used in the docking studies. The enzyme preparation and the hydrogen atoms addition was performed in the preparation process. The whole EGFR enzyme was defined as a receptor and the site sphere was selected on the basis of the binding location of WZ4002. By moving WZ4002 and the irrelevant water, molecule DY3002 was placed. The binding interaction energy was calculated to include Van der Waals, electrostatic, and torsional energy terms defined in the tripos force field. The structure optimization was performed using a genetic algorithm, and only the best-scoring ligand protein complexes were kept for analyses. The WWW site also includes many resources for use of AutoDock, including detailed tutorials that guide users through basic AutoDock usage, docking with flexible rings, and virtual screening with AutoDock. Tutorials may be found at: http://autodock.scripps.edu/faqs-help/tutorial.

## 4. Conclusions

In conclusion, we report the novel mutant selective inhibitor DY3002 that targets both sensitizing mutations and T790M resistance mutation, while leaving the wild-type form of the receptor unaffected. These explorations revealed DY3002 to effectively inhibit the EGFR^T790M/L858R^ enzyme at a surprisingly low concentration (0.71 nM), while having moderate activity (IC_50_ = 448.7 nM) against wild-type EGFR. Compared to rociletinib (SI = 21.4) and osimertinib (SI = 40.9), DY3002 significantly increased selectivity (SI = 632.0). In cell-based tests, DY3002 markedly inhibited the proliferation of H1975 cells, with an IC_50_ value of 0.037 μM. Moreover, the AO/EB and DAPI staining assays, and flow cytometer analyses indicated superior biological properties of DY3002 compared to references. An in vivo test in rat oral glucose tolerance model, revealed no hyperglycemia side effects of DY3002 via administering high drug doses (50 mg/kg). In summary, we identified the novel molecule DY3002, which serves as a promising EGFR^T790M^ inhibitor with the potential to overcome NSCLC resistance.

## Figures and Tables

**Table:** 

Time (h)			0	0.5	1	1.5	2	3	4	6	8	12
Blood Glucose (mmol/L)	Blank	1	5.7	5.6	7.0	6.5	5.2	5.7	4.9	5.2	4.7	4.6
		2	5.9	6.4	5.9	6.1	6.7	5.4	6.0	6.4	4.8	4.7
		3	5.9	6.1	5.5	5.4	6.3	5.9	5.2	5.6	5.0	5.7
	DY3002	1	6.2	5.9	6.3	6.0	4.8	5.4	4.8	5.5	6.3	5.8
		2	5.8	5.6	5.7	5.4	5.8	5.6	6.0	4.9	5.9	5.7
		3	5.5	6.0	4.8	4.6	5.6	5.2	5.4	5.1	3.9	4.9

**Table 1:** In vitro EGFR tyrosine kinases (wild-type and L858R/T790M mutation) activities ^a^.

Compounds	EGFR (IC_50_, nM) ^b^		SI(WT:L858R/T790M)
	WT	L858R/T790M	
DY3002	448.7	0.71	632.0
Rociletinib	460.2	21.5	21.4
Osimertinib	482.3	11.8	40.9
Gefitinib	15.5	823.3	--

^a^ Data represent the mean of at least three separate experiments; ^b^ Concentration needed to inhibit the autophosphorylation of the cytoplasmic domain of EGFR by 50%, as calculated using GraphPad Prim version 5.0 (GraphPad Software, Inc., La Jolla, CA, USA).

**Table 2:** Cellular antiproliferative activities ^a^.

Compounds	Cellular Antiproliferative Activity (IC_50_, μM) ^b^							
	H1975	HCC827	A431	H1299	LoVo	A549	LO-2	HBE
DY3002	0.037	0.0104	0.382	4.12	2.46	4.45	4.24	>40.0
Rociletinib	0.137	0.0181	1.29	20.1	20.1	6.50	8.57	>40.0
Osimertinib	0.087	0.0102	0.915	7.49	4.85	7.28	4.56	8.39
Gefitinib	10.8	0.0106	3.30	10.8	13.2	29.4	14.5	23.8

^a^ Data represent the mean of at least three separate experiments; ^b^ The IC_50_ values are the concentrations in micromolar needed to inhibit cell growth by 50% as calculated using GraphPad Prim version 5.0.
